# Australian Notes

**Published:** 1916-07-22

**Authors:** 


### AUSTRALIAN NOTES.

### CAUSES OF INVALIDITY.

The Committee of experts appointed in January to inquire into the causes of invalidity and death in Australia—Professor Sir Harry Allen (Melbourne University), Dr. Compston (Director Federal Quarantine), and Dr. A. Jeffreys Wood, of Melbourne—has presented its preliminary report to the (Federal) Minister for Customs (Mr. Tudor). The Committee so far has been examining statistics concerning the comparative frequency of occurrence and strength of common diseases in certain parts of the Commonwealth among both sexes at varying ages. This has revealed a number of interesting facts whose explanation will be largely the future work of the Committee. For example, why was the Melbourne metropolitan death-rate in 1914 12.21 per thousand, while Stawell, a small country town, showed 17.80, Ballarat and suburbs 17.51, and Bendigo and suburbs 16.46? Again, why did the death-rate in "Greater" Melbourne vary from 8.56 in Prestonshire and 9.60 in Malvern City to 14.76 in Melbourne City? The report states the problems with which the Committee finds itself confronted. Climate, soil, housing and ventilation, food, water supply, drainage, disposal of refuse, exercise, and social habits are factors that have to be kept in mind, and it is pointed out that care for the teeth, the throat, and the organs of digestion has a value that increases with knowledge.

Concerning the higher death-rates of Ballarat and Bendigo, an Australian would at once point out that both are mining centres with workers liable to accidents, Ballarat having a very cold winter climate and Bendigo a very mild one, while Prestonshire is a rather thinly settled farming district and Malvern a scattered residential area with wide spaces of parks, gardens, and vacant blocks. Stawell, though now a vineyard and orchard district, also has a gold-mining district adjacent. Further reports from the Committee are awaited with much interest.

### THE HOSPITALS.

At a recent meeting of the Melbourne Hospital Committee several important matters were brought up, the most insistent being the finances. The total deficit on March 14 was £18,529, compared with £14,447 on June 30, 1915. Urgent representations to the State Treasurer will probably be made at an early date. In face of this, a letter was received from the Medical Students' Society urging that an increase in the salaries paid to the hospital resident medical staff should be made, and pointing out that at the Alfred Hospital the "residents" receive £3 a week. The Chairman (Mr. John Grice) remarked that the students did not come to the hospital for what they received in the way of salary, but for experience, which was worth many times as much as they would receive if they acted as *locum tenentes* to outside practitioners. On the same date there were four cases of meningitis in the hospital—there because there is nowhere else for them to go—and six cases of phthisis. It was suggested that the attention of the Minister for Public Health should be directed to this fact in the hope that something might be done to relieve the institution of such patients. Dr. McMeekin (medical superintendent) stated that the hospital was treating cases of phthisis and general debility which ought to go to other institutions, and thereby depriving others, to whom permanent benefit might result, of hospital accommodation. It was decided that a statement should be prepared of the number of phthisis and other incurable cases accommodated in the Melbourne Hospital during the last six months. Affairs at Fairfield Infectious Diseases Hospital are improving. Plans provide for an ultimate extension of 600 beds. More ground is to be supplied by taking in part of the Yarra Bend Asylum grounds which adjoin the hospital. It is also intended to provide at some future date accommodation for 100 meningitis patients, fifty beds to be proceeded with at once, the Minister for Health hoping that the structure will be ready for occupation in about four months.

### MENINGITIS.

Several deaths have occurred from cerebro-spinal meningitis, and it speaks well for the preventive measures at the various military camps that the majority of the victims are civilians. Early in March the Minister for Health appointed a Medical Committee to draw up regulations for dealing with meningitis patients in order to prevent the dissemination of the disease. This Committee has produced a list of nine precautionary regulations which have been adopted by the Board of Health, and according to Dr. Robertson (chairman), the whole onus of administering these regulations will lie on the municipalities, but with power to recover the cost of treatment from any patient. The large municipality of Prahran promptly decided that meningitis was distinctly a national matter, and that owing to the large expenditure that would certainly be involved the whole scheme was so far removed from a municipal standpoint as to be unworkable. It was sugggested that joint municipal action against these regulations should be taken. This was agreed to. It is, of course, understood that the incidence of these regulations for checking the spread of meningitis on the municipalities is only temporary, pending the appointment of local and independent health officers.

### HEALTH ADMINISTRATION.

Under the new Health Bill in charge of the (State) Minister for Health for Victoria (Mr. McLeod), a complete change in the system of administration is anticipated. For a long time it has been recognised that the present Board of Public Health, with its antiquated machinery, has outlived its usefulness, and last year a proposal was under consideration to substitute for the Board a council of experts, under the Minister. The constitution of the Central Authority has not yet been decided upon, but it is certain that it will be very different from the present controlling body, which is chiefly composed of representatives of the municipalities. On account of interests involved, Mr. McLeod is not in favour of municipal councils as health authorities; he favours the appointment of district health officers salaried by the Government; they will be responsible only to the Central Authority, and they will be required to undertake vaccination and some other tasks besides the work performed by the present (municipal) health officers, and their time will be too full to permit of them undertaking private practice.

